# An innovative cell model revealed the inhibitory effect of flavanone structure on peroxynitrite production through interaction with the IKKβ kinase domain at ATP binding site

**DOI:** 10.1002/fsn3.1591

**Published:** 2020-04-27

**Authors:** Supochana Charoensin, Tzou‐Chi Huang, Jue‐Liang Hsu

**Affiliations:** ^1^ Department of Tropical Agriculture and International Cooperation National Pingtung University of Science and Technology Pingtung Taiwan; ^2^ Department of Biological Science and Technology National Pingtung University of Science and Technology Pingtung Taiwan

**Keywords:** antioxidant activity, cell model, chemical structure, flavanone, macrophage, peroxynitrite

## Abstract

It is hypothesized that the oxidative/nitrosative stress inhibitory effect of a flavanone is governed by its chemical structure. However, the existing cell‐based antioxidant assays primarily focus on single chemical to initiate toxic species production. In this study, a novel cell model using macrophage treated with a combination of PMA and LPS leading to generation of peroxynitrite was proposed to provide a more real physiological condition. Three flavanones (eriodictyol, naringenin, and pinocembrin) with different number of ortho‐dihydroxyl groups on B‐ring were used to provide a more comprehensive evaluation of the role of chemical structure in the new model. Dihydrorhodamine123 assay, protein immunoblotting, immunofluorescence assay, and in silico analysis by molecular docking between the flavanones and IKKβ catalytic kinase domain at the ATP binding site were employed. Results indicated that the generation of peroxynitrite was decreased at 10 µM of flavanones; eriodictyol was the most effective inhibitor. Western blot analysis and confocal fluorescence image also showed that eriodictyol could inhibit iNOS and p47 protein expressions through the inhibition of NF‐kB translocation and performed the maximal inhibition compared to that of the other groups. In addition, the highest CDOCKER energy values of eriodictyol (38.6703 kcal/mol) confirmed that the 3′,4′‐ortho‐dihydroxylation on the B‐ring played a crucial role in binding with IKKβ kinase domain at ATP binding site. Finally, we propose that the ortho‐dihydroxyl groups on B‐ring of flavanone may influence directly the occupation of the ATP binding site of IKKβ kinase domain leading to the abrogation of peroxynitrite formation in the innovative cell model.

## Graphical abstract

The roles of three flavonoids (eriodictyol, naringenin, and pinocembrin) in peroxynitrile inhibition and their interactions with ATP binding site of the kinase domain of IKKβ were comprehensively studied. The ortho‐dihydroxyl groups on B‐ring of flavanone may influence directly the occupation of the ATP binding site of IKKβ kinase domain leading to the abrogation of peroxynitrite formation in the innovative cell model.

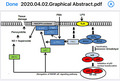

## 1 INTRODUCTION

It is evidenced that free radicals, both reactive oxygen (ROS) and nitrogen species (RNS), play a dual role in both causing oxidative/nitrosative stress, tissue damages, and regulating various physiological functions that are beneficial to the living system (Lushchak, 2014). In fact, excess production of free radicals can cause the stresses that lead to many human diseases, such as Alzheimer's disease, diabetes mellitus, cardiovascular diseases, asthma, and cancer (Bender & Graziano, 2015; Di Meo, Reed, Venditti, & Victor, 2016). Macrophage has been proven to play an important role in regulating the immune system in response to chronic, autoimmune, and infectious diseases (Navegantes et al., 2017). However, their overexpression can induce tissue damage during infection, autoimmune, and chronic diseases. Indeed, the activated macrophages release several toxic species (nitric oxide, superoxide, and MMP), cytokines, and enzymes at the site of inflammation or infection, leading to the activation of numerous signaling cascades that promote the expression of proinflammatory factors. It has been determined that chronic diseases showed the coincidence of low‐grade inflammatory factors and imbalance in ROS production (Biswas, 2016).

It has been proposed that the simultaneous activation of superoxide ( $O_{2}^{-}$ ) and nitric oxide (NO) synthesis within a cell induces their diffusion‐controlled reaction to produce peroxynitrite (ONOO^−^), which is a powerful oxidant against the invading agents (Koppenol, 2001; Padmaja & Huie, 1993). In activated macrophages, ONOO^−^ may be an important mediator of free radical‐induced cellular injury. Recently, ONOO^−^ has also been recognized to be a potent macrophage‐derived cytotoxin (Prolo, Álvarez, & Radi, 2014). The overproduction of ONOO^−^ can adversely affect cell function and mode of cell death, culminating in tissue injury and inflammatory diseases (such as myocardial ischemia, diabetes, cancer, and cardiovascular disorders) (Pacher, Beckman, & Liaudet, 2007; Virág, Szabo, Gergely, & Szabo, 2003). Until now, however, all of the macrophage cell models were focused on single chemical to evaluate the effect of antioxidants on the inhibition of $O_{2}^{-}$ or NO formation (Chao, Hong, Chen, & Lin, 2010; Kongpichitchoke, Hsu, & Huang, 2015; Yang, Ham, & Choi, 2013), and no cell model was developed to investigate the mechanism of tissue damage caused by ONOO^−^ formation from reaction of $O_{2}^{-}$ and NO, that is, close to the real physiological condition.

**FIGURE 1:**
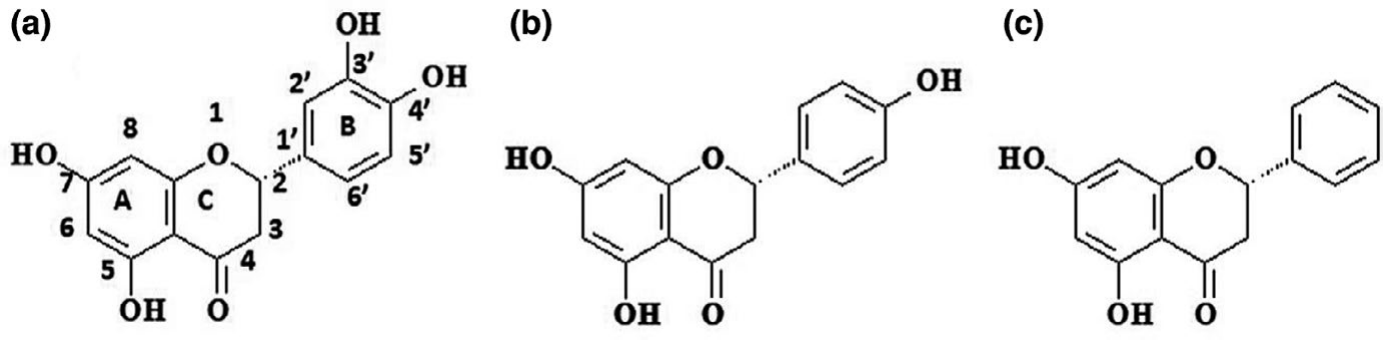
Molecular structure of flavanones. Eriodictyol (a), naringenin (b), and pinocembrin (c)

The peroxynitrite‐scavenging ability was previously defined by the attenuation of peroxynitrite‐mediated tyrosine nitration (Ho, Su, & Lin, 2014; Pannala, Rice‐Evans, Halliwell, & Singh, 1997). The results indicated a high correlation between the total phenolics and the peroxynitrite‐scavenging activity. In fact, flavonoids, one of the main classes of polyphenols, are responsible for many benefits of medicine plants, especially antioxidant (Magiera, Baranowska, & Lautenszleger, 2015), antimicrobial (Akhavan, Jahangiri, & Shafaghat, 2015), anticancer (Ravishankar, Rajora, Greco, & Osborn, 2013), antidiabetic (Babu, Liu, & Gilbert, 2013), and anti‐inflammatory activities (Zhou, Lutterodt, Cheng, & Yu, 2009). Eriodictyol 7‐rutinoside, naringenin 7‐O‐rutinoside, and pinocembrin ‐7‐O‐ feruloyl glucoside, three closely structure‐related compounds with different number of ortho‐dihydroxyl groups on the B‐ring (two, one, and zero, respectively—Figure 1), were found to be the major antioxidative flavonoid glycosides in lemon fruit (Miyake, Yamamoto, Morimitsu, & Osawa, 1998), and a variety of plants, including *Pinus* heartwood, *Eucalyptus*, *Populus*, *Euphorbia*, and *Sparattosperma leucanthum* (Rasul et al., 2013). In the present study, therefore, these compounds were used to illustrate the relationship between the structure and peroxynitrite‐scavenging activities of flavanones in an innovative cell model.

## 2 MATERIALS AND METHODS

### 2.1 Chemicals

Dulbecco's modified Eagle's medium (DMEM) was purchased from Gibco BRL. 3‐(4,5‐dimethylthiazol‐2‐yl)‐2,5‐diphenyltetrazolium bromide (MTT) were obtained from AppliChem. PMA, LPS, DHR123, DMSO, Eriodictyol, Naringenin, and Pinocembrin were bought from Sigma‐Aldrich. Fetal bovine serum (FBS) and antibiotics were supplied by Biological Industries.

### 2.2 Cell culture and treatments

Macrophage RAW264.7 cells were obtained from Bioresource Collection and Research Center and grown in a 10 cm Petri dish containing DMEM supplemented with 10% FBS and 0.5% antibiotics (v/v). The cell dishes were maintained at 37°C, 5% CO_2_, and 95% air in a humidified incubator and subcultured every 2 days. Cells in culture medium were then plated into 96‐well (4 × 10^4^ cells/well) or 6‐well plates (1 × 10^6^ cells/well) and incubated at the same conditions before the flavanone treatments.

### 2.3 Cell viability assay

Cytotoxic effects of the flavanones were investigated by MTT assay. In short, the cells in 96‐well plates were incubated for 24 hr before treated with different concentrations of each flavanone (10, 20, 40, 80, and 100 µM) for 24 hr in 10 replicates. Subsequently, the medium was removed prior to addition of MTT into each well. After 3 hr of incubation at room temperature, the formanzan compound was dissolved by DMSO for 30 min. Finally, the absorbance was measured at 570 nm using VersaMax microplate reader (Molecular Devices LLC). The experiment was repeated three times.

### 2.4 Peroxynitrite determination

Measurement of peroxynitrite‐induced oxidation of dihydrorhodamine 123 was adapted from the literature (Mahajan, Chandra, Dave, Nanduri, & Gupta, 2012). A total of 1 × 10^6^ cells/well was seeded in 6‐well plates for 12 hr, and each well was then treated with each flavanone (10 μM) for 24 hr, followed by addition of LPS (1 µg/ml) plus PMA (100 ng/ml) for 6, 12, and 24 hr before the end of incubation to induce peroxynitrite production. After treatment, the cells were washed twice in PBS and centrifuged (500 *g*, 4°C, 5 min). The pellet was then incubated in darkness with 10 μM of DHR123. After 30 min of incubation at 37°C, fluorescence intensity of DHR123 was measured with a FACSCalibur™ flow cytometer (BD Biosciences). Dot plot under E‐1 mode was used for area gating (location of >90% cells). Data were collected from 10,000 gated cells.

### 2.5 Western blot analysis

RAW264.7 cells were separately pretreated with each of flavanone at the final concentration of 10 μM for 24 hr prior to 30 min of stimulation with LPS (1 µg/ml) and PMA (100 ng/ml). The stimulated cells were then collected to quantify the total proteins using BCA protein assay kit (Thermo Scientific), while bovine serum albumin was used as standard. Thirty microgram of protein per sample was loaded onto 8% SDS–polyacrylamide gel to separate proteins. Proteins from the gel were transferred into a PVDF membrane (PerkinElmer) at 250 mA for 1 hr, followed by blotting with primary target antibodies (Cell Signaling), β‐actin (cat. no. 4970), iNOS (cat. no. 13120), and p47 (cat. no. 4312), at final dilution of 1:1,000 at 4°C. After 16–18 hr, the membranes were blocked by TBST buffer (Tris‐buffered saline, 0.1% Tween 20) with 1% BSA for 1 hr and then incubated at room temperature with secondary antibodies (Goat Anti‐Mouse IgG Antibody, (H+L) HRP conjugate, AP308P, Merck Millipore) at a dilution of 1:5,000 for 1 hr. After 1 hr incubation with TBST buffer, the protein bands were subjected to chemiluminescence reagent (ECL, Advansta) and photographed with a Gel image system.

### 2.6 Immunofluorescence microscopy

RAW264.7 cells at the density of 1 × 10^5^ cells/well were seeded in a 12‐well plate and were pretreated with 10 μM of eriodictyol for 24 hr, followed by incubation with LPS (1 µg/ml) and PMA (100 ng/ml) for 30 min. After treatments, the cells were fixed with 4% paraformaldehyde solution for 30 min, permeabilized in 0.1% Triton X‐100 for 5 min, and blocked with 5% BSA for 1 hr at room temperature. The fixed cells were further incubated with anti‐NF‐κB (p65) (sc‐8008, Santa Cruz Biotechnology) antibody for 2 hr, followed by washing with PBST 3 times. The specimens were then stained 1 hr with the second antibody goat anti‐mouse IgG (Alexa Fluor^®^ 594, cat. no. A32740, Invitrogen) and counterstained with 0.5 mg/ml of DAPI solution for 10 min at room temperature. The localization of NF‐κB was imaged using a CARV confocal imager (BD Biosciences) as described previously (Chen, Tillberg, & Boyden, 2015).

### 2.7 Molecular docking

The potential docking was performed using Accelrys Discovery Studio Visualizer version 3.0. Proteins and molecular ligands data were obtained from RCSB (PDB code 3RZF) and NCBI databases, respectively. Water molecules and ligand were removed from the protein structure, while CHARMM program was used to minimize molecules' energy before docking. Binding site of kinase domain was defined by using Leu‐26, Val‐29, Ala‐42, Met‐96, Glu‐97, Tyr‐98, Cys‐99, Asp‐166, Leu‐167, and Gly‐168 coordinate (*X*, 90.992; *Y*, −27.239; and *Z*, 53.945). In CDOCKER tool viewer setting, the docking process was optimized to obtain the best 10 poses based on ‐CDOCKER_energy and sequentially visualize ligand–receptor interactions through hydrogen bonds.

### 2.8 Statistical analysis

Experimental results were expressed as the mean ± standard deviation (*SD*). SPSS 22.0 statistical software was used for statistical analysis. The significance of differences between the treatments was determined by analysis of variance, followed by Duncan's multiple range test or *t* test (*p* < .05).

## 3 RESULTS AND DISCUSSION

### 3.1 Cytotoxicity of eriodictyol, naringenin, and pinocembrin in RAW264.7 cells

**FIGURE 2:**
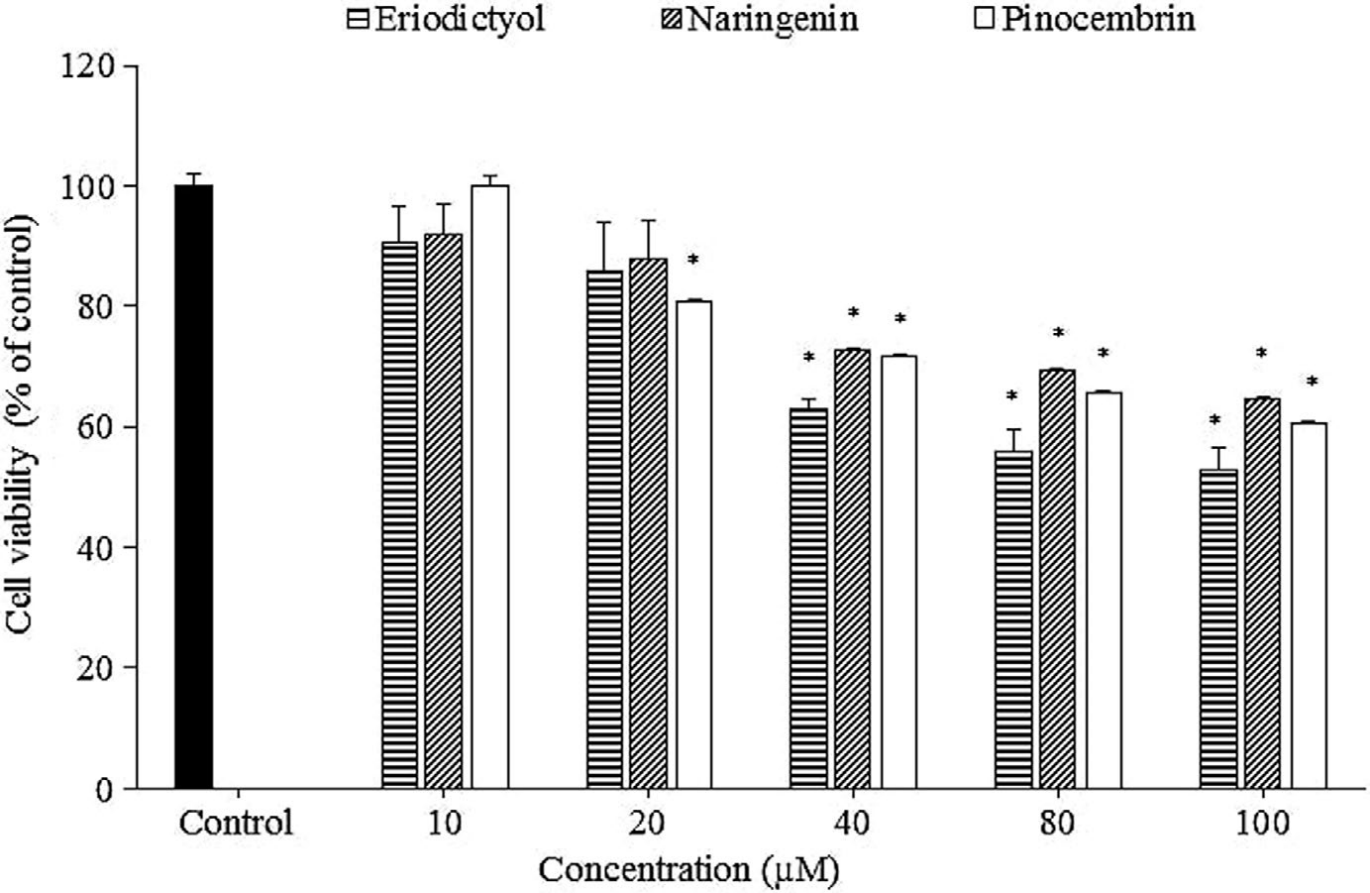
Cytotoxicity of eriodictyol, naringenin, and pinocembrin in RAW 264.7 macrophage cells. Macrophage RAW264.7 cell was treated with 0–100 µM of flavanones for 24 hr. The experiments were performed with 6 replicates. The results are expressed as mean ± *SD*. Asterisks (*) indicate statistical differences between each treatment and control group by Student's *t* test (*p* < .05)

It was found that eriodictyol, pinocembrin, and naringenin at 10 μM did not significantly affect the viability of RAW264.7 cells after 24 hr of exposure (Figure 2). At the concentration of 20 μM, the cell viability of RAW264.7 cells was not affected by eriodictyol and naringenin. However, relatively high cytotoxicity on RAW264.7 cells was observed in the other tested concentrations, especially cell death was observed at 100 μM. The result of pinocembrin was similar to those regarding its cytotoxicity in Y‐79 cell (Chen, Shi, Chien, & Shih, 2014), suggesting that the toxicity of pinocembrin is not dependent on the types of cell lines. However, a quite low cytotoxic concentration of eriodictyol and naringenin was observed in this study (40 μM). This finding is inconsistent with the results reported by Huang et al. (2013), where eriodictyol did not show considerable cytotoxicity at 100 μM in U937 cells, or by C.‐L. Chao, Weng, et al. (2010) in RAW264.7 and microglia cells, where the cell viability was only affected at the high concentrations of naringenin (300 and 200 μM, respectively). Therefore, the cytotoxic data of flavanones are very necessary and should be established prior to conducting any experiment. Further, it is suggested to select the 10 μM concentration throughout this experiment.

### 3.2 Peroxynitrite inhibition by flavanones in the new cell model

**FIGURE 3:**
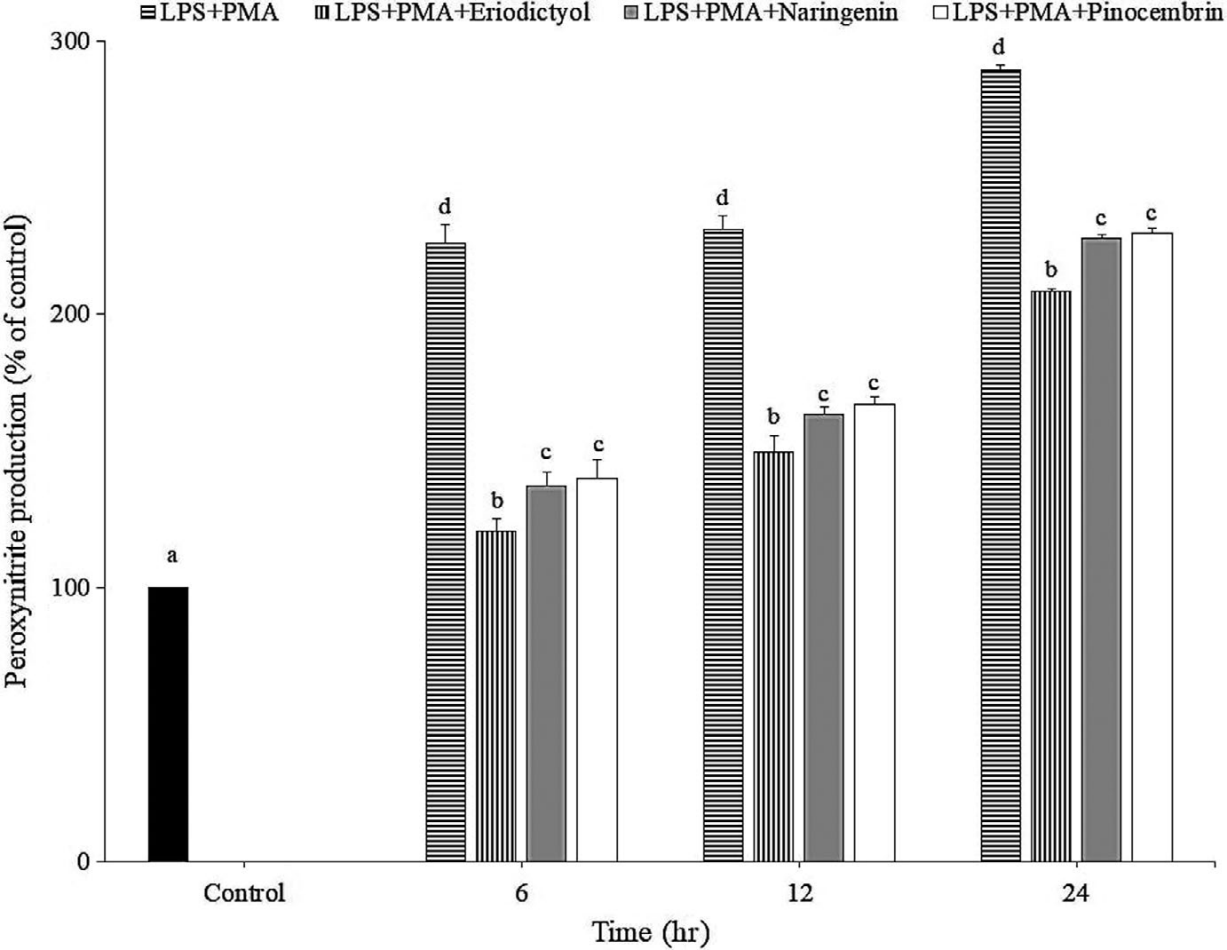
Effect of flavanones on peroxynitrite reduction in LPS plus PMA‐activated RAW264.7 cell. Cells were treated with the flavanones for 24 hr, followed by stimulation with LPS (1 µg/ml) and PMA (100 ng/ml) for 6, 12, and 24 hr before the end of incubation. Cells without flavanones treatment followed by stimulation LPS and PMA were named as positive control. The results were expressed as mean ± *SD* of three replicates. Values within the same time having different superscripts are significantly different (*p* < .05, Duncan test)

The oxidation of dihydrorhodamine 123 to fluorescent rhodamine 123 in macrophage was used to measure the production of peroxynitrite. Upon combined stimulation with LPS (1 µg/ml) and PMA (100 ng/ml), a significant increase in 123‐rhodamine formation compared to unstimulated macrophages was detected during 24 hr (Figure 3). Interestingly, peroxynitrite production was markedly inhibited in the cells treated with eriodictyol, naringenin, or pinocembrin at 10 μM. The highest inhibitor was eriodictyol, followed by naringenin and pinocembrin at all times, implying that their activities were not time‐dependent. It is also well known that the ortho‐dihydroxyl groups of ring B are responsible for the radicals scavenging capability of flavonoids (Williams, Spencer, & Rice‐Evans, 2004). However, the presence of one OH group in ring B only offers a little advantage for the antioxidative activity of flavonoids (Rice‐Evans, Miller, & Paganga, 1996). Thus, it is reasonable that naringenin did not decrease significantly peroxynitrite production compared to pinocembrin. Our previous study also demonstrated that, in PMA‐stimulated RAW264.7 cells, eriodictyol at 10 μM significantly suppressed (63.4%) the phosphorylation of Protein kinase C delta (PKCδ), whereas the inhibitory properties of naringenin and pinocembrin were exhibited at 72.6% and 76.8%, respectively. In fact, PKCδ phosphorylates the regulatory subunits p47phox leading to the assembly of NADPH oxidase complex for $O_{2}^{-}$ generation. As a result, eriodictyol inhibited the formation of $O_{2}^{-}$ and this greatly contributed to the antioxidant capacity of eriodictyol (Kongpichitchoke et al., 2015). Hence, this study confirmed the ability of eriodictyol, naringenin, and pinocembrin to inhibit peroxynitrite formation, and suggested a more accurate and susceptible model to investigate the mechanisms of peroxynitrite‐mediated formation of rhodamine.

### 3.3 Inhibition of iNOS and p47 by flavanones leads to reduced peroxynitrite production

**FIGURE 4:**
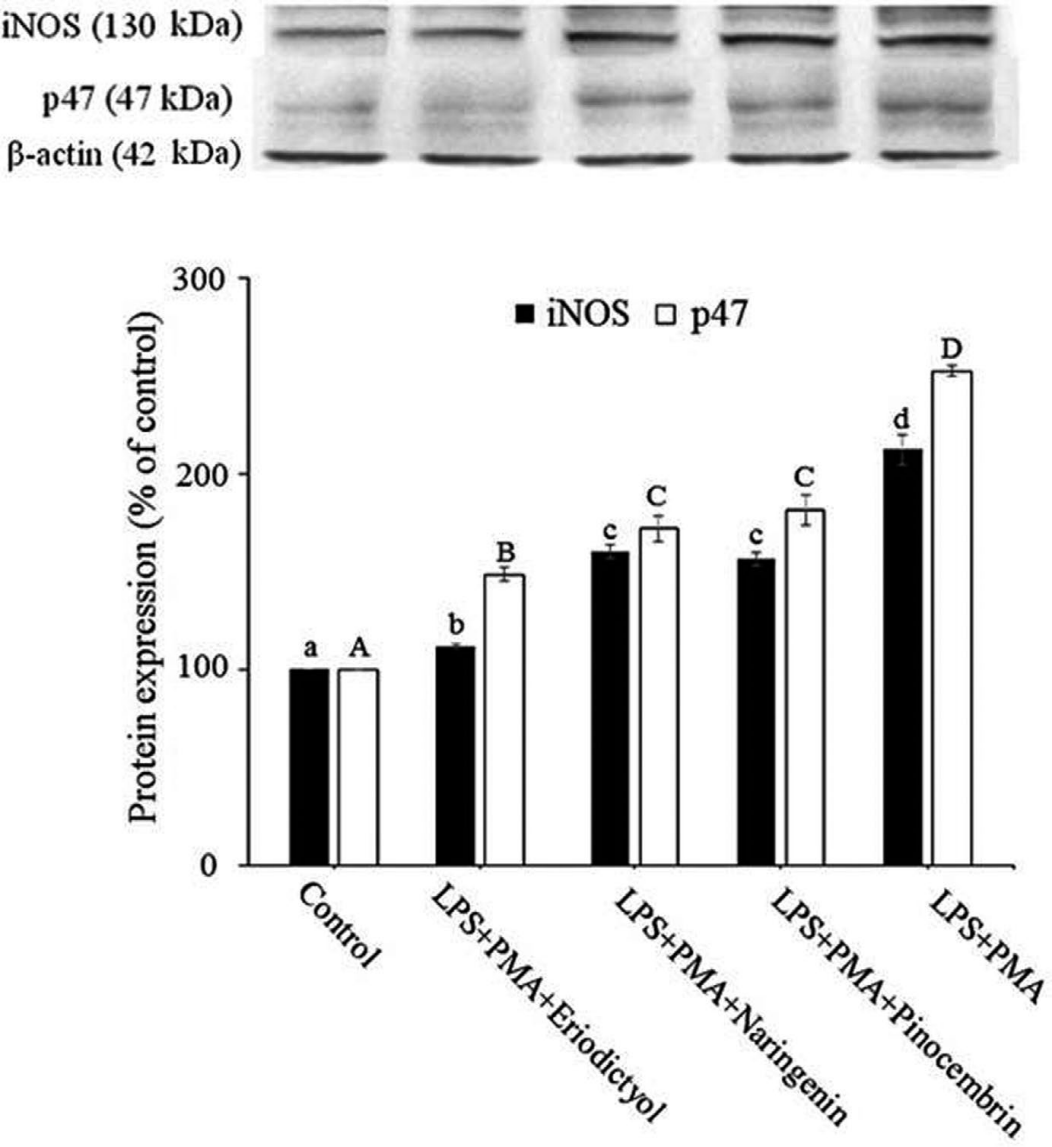
Inhibitory effect of flavanones on iNOS and p47 expressions in RAW 264.7 cells. RAW 264.7 cells were pretreated with eriodictyol, naringenin, or pinocembrin (10 μM) for 24 hr prior to stimulation with LPS plus PMA (30 min). The results were expressed as mean ± *SD* of three replicates. Different letters indicate the significant difference between groups (*p* < .05, Duncan test)

It has been determined that macrophages produce ROS and RNS, including $O_{2}^{-}$ and NO, that not only trigger the immune responses against pathogens, but also induce tissue destruction and lead to chronic inflammation (Cheng, Wang, Fu, & Xu, 2019; Nathan & Ding, 2010). In which, NO is commonly synthesized by iNOS and then reacts directly with $O_{2}^{-}$ to yield ONOO^−^, which triggers the formation of nitrotyrosine, which indirectly improves antimicrobial and cytotoxic activities of macrophages (Xia & Zweier, 1997). It is demonstrated that naringenin inhibited the expression of iNOS and NADPH oxidase in LPS‐stimulated murine macrophages through AMPK‐ATF3‐dependent negative regulation of the LPS/TLR4 signaling pathway (Liu et al., 2016). On the other hand, NADPH oxidases in the activated macrophages are an important source of $O_{2}^{-}$ in response to pathogen invasion (Tan et al., 2016). Previous studies showed that PMA‐treated RAW264.7 cells promoted the translocation of phosphorylated p47 to the membrane and interaction with membrane‐bound components to form the active NADPH oxidase assembly (Kongpichitchoke et al., 2015), while LPS‐stimulated macrophage induces the production of various proinflammatory mediators, as well as excess NO formation via iNOS overexpression (Castaneda, Lee, Ho, & Huang, 2017). In our study, as shown in Figure 4, the flavanones significantly decreased iNOS and p47 expressions in the LPS plus PMA‐stimulated RAW264.7 cells. This finding indicated that the inhibitory effect of plant‐derived flavonoid glycosides on ONOO^−^ production is attributed to their ability to reduce iNOS and p47 formation. In addition, the activation of NADPH oxidase in macrophage was demonstrated to initiate after the phosphorylation of p47 induced by PMA (Liu et al., 2015), while iNOS is readily stimulated in macrophages by LPS (Aldridge, Razzak, Babcock, Helton, & Espat, 2008; Stuehr & Nathan, 1989), suggesting a more comprehensive and precise mechanism integrated with our new cell model. Also, eriodictyol showed the maximal inhibition on both of proteins compared with naringenin and pinocembrin. Together with the results of Section 3.2, it is reasonable to presume that the inhibitory efficiency of flavanones was correlated with their number of ortho‐dihydroxyl groups on the B‐ring.

### 3.4 Eriodictyol abrogated IKK/NF‐κB signaling by blocking the nuclear translocation of NF‐κB

**FIGURE 5:**
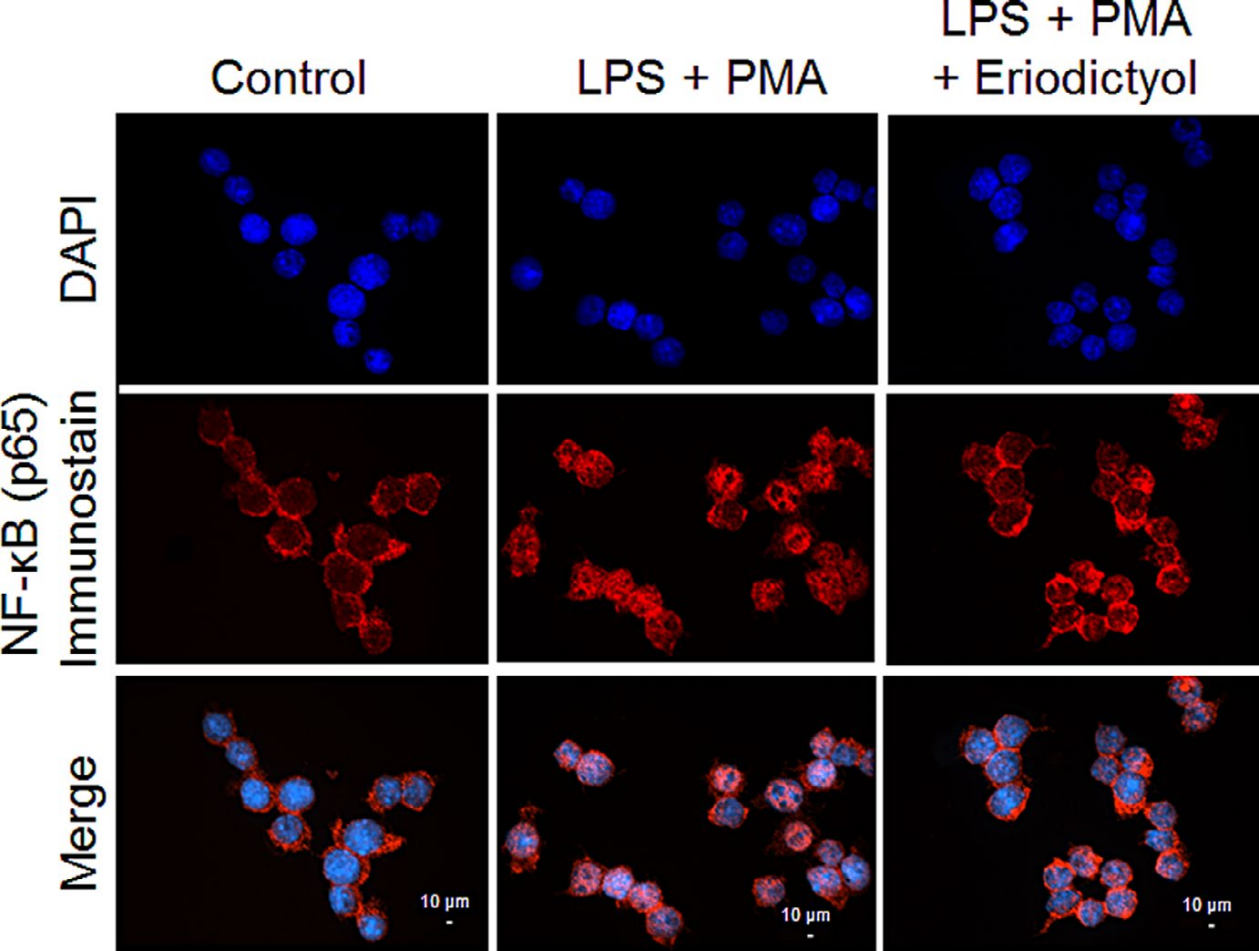
Effect of eriodictyol on NF‐κB (p65) nuclear translocation in cultured RAW264.7 cells. Cells were pretreated with 10 μM of eriodictyol for 24 hr, followed by incubation with LPS (1 μg/ml) and PMA (100 ng/ml) for 30 min. Cells were then stained with p65 antibody, Alexa Fluor 594 goat anti‐mouse (red) and counterstained with DAPI (blue). Scale bar: 10 µm. Tests were performed in triplicate

Under stimulatory conditions, IκB kinase (IKK) phosphorylates the inhibitory subunit (IκB) leading to successive degradation of IκB proteins that grants the NF‐κB nucleus translocation, and binding reactions with the κB site (Hayden & Ghosh, 2004). In the present study, to elucidate the mechanism of IKK/NF‐κB inactivation by eriodictyol, the protein expression of NF‐κB (p65) was analyzed by immunofluorescence microscopy. In which, NF‐κB p65 was labelled with a red fluorescence to track the influence of eriodictyol on its translocation. Fluorescence analysis with confocal microscope revealed that eriodictyol inhibited the p65 translocation into the nucleus. As shown in Figure 5, NF‐κB p65 translocation increased 30 min after LPS (1 μg/ml) and PMA (100 ng/ml) administration. Treatment of the macrophage cells with 10 μM eriodictyol for 24 hr resulted in a significant reduction of NF‐κB translocation from the cytosol into nucleus. It has been proven that the IKK complex contains two subunits (IKKα and IKKβ) and a regulator (IKKγ). Numerous studies also suggested a central role of IKKβ in the inflammatory stimuli‐induced activation of NF‐κB and demonstrated that IKKβ plays a more important role in NF‐κB signaling (Ghosh & Karin, 2002; Li et al., 1999; Tanaka et al., 1999). Moreover, it has been proposed that pinocembrin, the major active compound in the roots of *Alpinia pricei Hayata*, decreased LPS‐induced NO and ROS production through the inhibition of NF‐κB nuclear translocation (Yu, Hsu, & Yen, 2009). Therefore, we postulated that the ability of eriodictyol to inhibit the NF‐κB translocation was similar to that of pinocembrin and governed by the inactivation of IKKβ in IKK/NF‐κB signaling pathway, leading to a reduction in the protein expressions of iNOS and p47.

### 3.5 Binding mechanism of the flavanones to kinase domain of IKKβ

**FIGURE 6:**
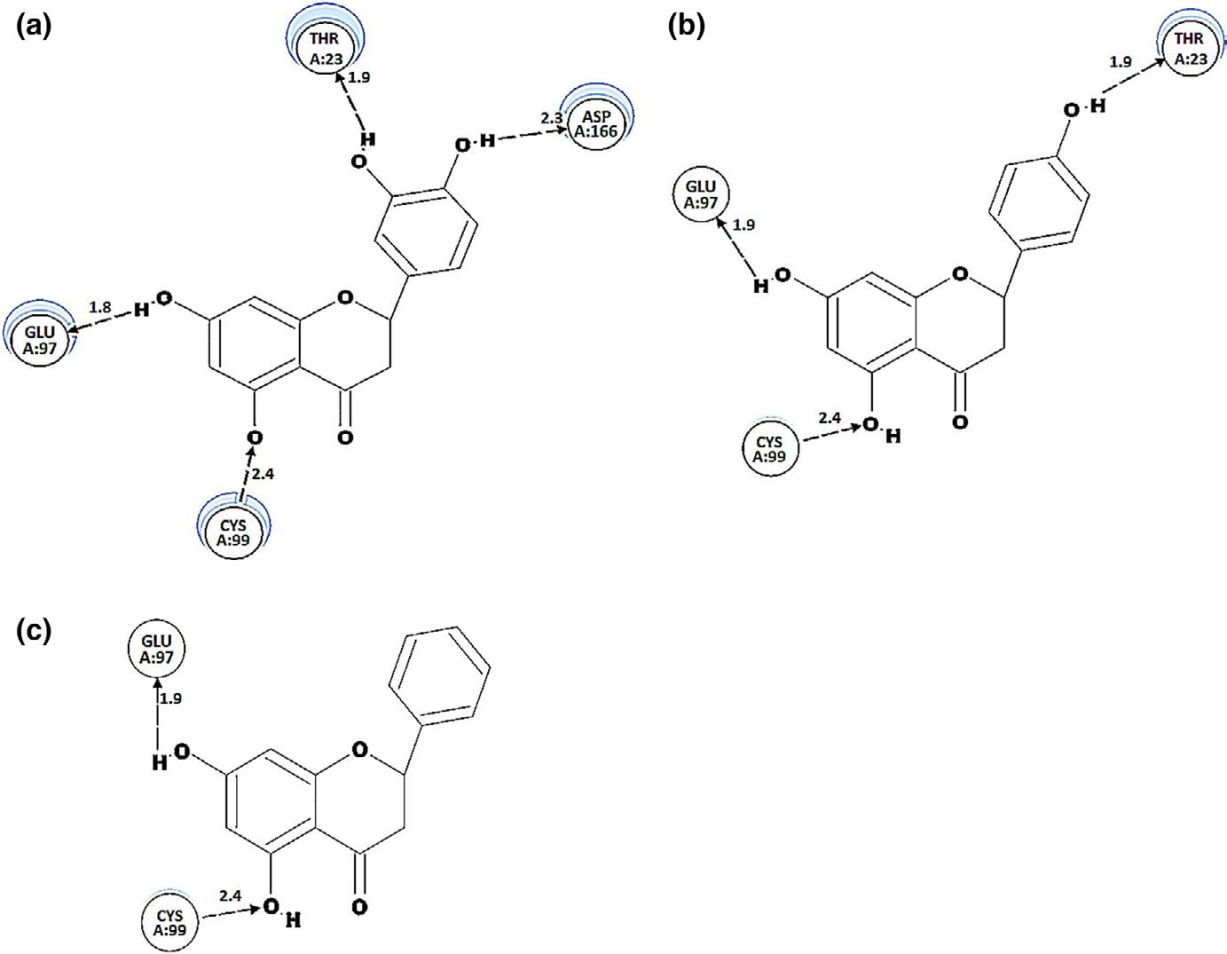
Docking simulation showing the hydrogen bond interaction of flavanones with the X‐ray crystal structure of kinase domain amino acid of IKKβ at ATP binding site from ‐CDOCKER_energy values. Numbers indicate hydrogen‐bonding lengths. Image generated using Accelrys Discovery Studio Visualizer software. (a) Eriodictyol, (b) Naringenin, and (c) Pinocembrin

For a long time, several inhibitory molecules of IKKβ have been recognized based on their structure or using high‐throughput screening (Ma et al., 2012; Miller et al., 2011; Zhong et al., 2012). Nevertheless, the recent availability of IKKβ crystal structure allowed us to utilize molecular docking to identify the novel IKKβ inhibitors (Leung, Chan, Li, Fong, & Ma, 2013; Xu et al., 2011). Thus, in this study, this in silico method was used to delineate the inhibitory effects of flavanones on IKKβ. In brief, the binding site of IKKβ was defined to be within 3 Å of the bound inhibitor, which is located at the interlope connector linking the N and C lobes of the lKKβ kinase domain (KD) following a previous study (Leung et al., 2013). The major receptor residues in specifying kinase inhibitor in the crystal structure of IKKβ are Met‐96, Glu‐97, Tyr‐95, and Cys‐99. The backbone groups of Glu‐97 and Cys‐99 are able to provide hydrogen‐bonding interactions with the inhibitor (Xu et al., 2011). The binding site of IKKβ was defined at Leu‐26, Val‐29, Ala‐42, Met‐96, Glu‐97, Tyr‐98, Cys‐99, Asp‐166, Leu‐167, and Gly‐168 of the KD. It was applied as the ligand‐binding sites for docking computation in the current investigation. Computational simulation showed that the poses of eriodictyol, naringenin, and pinocembrin with docking scores of 38.6703, 32.9741, and 30.2863, respectively, were observed within the ligand‐binding IKKβ kinase domain, in which eriodictyol was the most favorable flavanone (Table 1). Moreover, Table 1 indicated different properties of hydrogen bonding with the IKKβ KD of the flavanones. It is visible that eriodictyol contained four hydrogen‐bonding bridges. The hydrogen atoms from 3′,4′‐ortho‐dihydroxyl on the B‐ring were bounded to Thr‐23 and Asp‐166 to create the first and the second bridges, while the third and fourth hydrogen bridges were formed between two OH groups in the ring A and Glu‐97 and Cys‐99 (Figure 6a). For naringenin, its interaction was made by forming three hydrogen bonds from 4′‐ortho‐dihydroxyl group on the B‐ring with Thr‐23. The second and third bridges were the same as the third and fourth hydrogen bond in eriodictyol (Figure 6b). Two hydrogen bonds of pinocembrin were formed between two hydrogen atoms of OH group at C7, C5 and Glu‐97, Cys‐99, respectively (Figure 6c). Even though a weaker hydrogen‐bonding parameter is known for B‐ring compared to A‐ring (Suresh, Srivastava, & Mishra, 2012), our results showed that the 3′,4′‐ortho‐dihydroxyl groups in the B‐ring of eriodictyol have an important role in inhibiting the formation of peroxynitrite by the formation of a strong hydrogen‐bonding interaction with the binding domain.

**TABLE 1:** Comparison of the hydrogen‐bonding characteristics between the pose of each flavanone with kinase domain of X‐ray crystal structure of IKKβ at ATP binding site (Radius 16.252, Coordinate *X*, 90.992; *Y*, −27.239; and *Z*, 53.945)

Ligands	‐CDOCKER_energy	Hydrogen bonding	
		Amino acid residue	Distance (Å)
Eriodictyol	38.6703	Thr‐23	1.9455
		Asp‐166	2.3022
		Glu‐97	1.8482
		Cys‐99	2.4010
Naringenin	32.9741	Thr‐23	1.9388
		Glu‐97	1.8772
		Cys‐99	2.4304
Pinocembrin	30.2863	Glu‐97	1.8871
		Cys‐99	2.3856

## 4 CONCLUSION

In summary, the stimulation with both LPS and PMA in new cell model was simple and more correlated with the real oxidative/nitrosative stress. This may help to gain a broader understanding of the way that ROS and RNS modulate cell function and may allow developing new strategies to monitor cell changes in many stress‐related diseases. Further, our results of examined three different flavanones expressed significant inhibitory effects on peroxynitrite production in the new cell model. Relationship between chemical structure and bioactivities of flavanones in the stimulated cells showed that the number of ortho‐dihydroxyl groups on the B‐ring regulated peroxynitrite inhibition by direct modulation of iNOS and p47 expressions. On the other hand, the difference in this group also remarkably affected the activity of eriodictyol, which has two hydrogen bridges, on binding with the ATP binding site of IKKβ kinase domain and the inhibition of NF‐kB translocation. We proposed that eriodictyol interacted with the binding site of IKKβ leading to the abrogation of IKK/NF‐κB signaling by blocking the nuclear translocation of NF‐κB. The decrease of iNOS and p47 protein expression resulted in the inhibition of nitric oxide and superoxide, respectively, and, subsequently, peroxynitrite formation in RAW 264.7 Cells.

## CONFLICT OF INTEREST

The authors declare that they have no conflict of interest.

## ETHICAL APPROVAL

The study did not involve any human or animal testing.
